# Liquid biopsy in ovarian cancer in China and the world: current status and future perspectives

**DOI:** 10.3389/fonc.2023.1276085

**Published:** 2023-12-19

**Authors:** Hui Zhang, Lingxia Wang, Huanwen Wu

**Affiliations:** ^1^ Department of Pathology, Peking Union Medical College Hospital, Chinese Academy of Medical Sciences and Peking Union Medical College, Beijing, China; ^2^ MRL Global Medical Affairs, MSD China, Shanghai, China

**Keywords:** ovarian cancer, liquid biopsy, circulating tumor DNA, cell-free DNA, poly(ADP)ribose polymerase inhibitors, China

## Abstract

Ovarian cancer (OC) is the eighth most common cancer in women, but the mild, non-specific clinical presentation in early stages often prevents diagnosis until progression to advanced-stage disease, contributing to the high mortality associated with OC. While serum cancer antigen 125 (CA-125) has been successfully used as a blood-borne marker and is routinely monitored in patients with OC, CA-125 testing has limitations in sensitivity and specificity and does not provide direct information on important molecular characteristics that can guide treatment decisions, such as homologous recombination repair deficiency. We comprehensively review the literature surrounding methods based on liquid biopsies, which may provide improvements in sensitivity, specificity, and provide valuable additional information to enable early diagnosis, monitoring of recurrence/progression/therapeutic response, and accurate prognostication for patients with OC, highlighting applications of this research in China.

## 1 Introduction

Worldwide, ovarian cancer (OC) was the eighth most common cancer and cause of cancer-related death in women in 2020, accounting for 1.6% of all new cancer cases and 2.1% of all cancer-related deaths (1). In China, more than 57,000 new cases of OC were reported in 2020, with over 39,000 deaths (2). More than 75% of OC is diagnosed at an advanced stage because early-stage ovarian tumors often present with mild, non-specific symptoms and minimal physical findings or may be asymptomatic (3, 4). Guidelines from the Society of Gynecologic Oncology (SGO) and the American Society of Clinical Oncology (ASCO) recommend ultrasonography, radiographic imaging, cancer antigen 125 (CA-125) serum level testing, and surgical biopsy (5).

Outcomes for patients with OC are strongly associated with disease stage at diagnosis. The 5-year overall survival (OS) rates are ~80%, ~60%, ~30%, and ~20% among patients with stage I, II, III, and IV OC, respectively (4). OC is also associated with high morbidity and high rates of relapse and metastasis, despite good responses to primary surgery and chemotherapy (6, 7). Therefore, several efforts have been made to establish tools for early diagnosis of OC. Tissue biopsy is considered standard for the histological diagnosis of OC (8), combined with imaging for staging. However, tumor biopsy is invasive and because of the non-specific symptomatology of OC, patients often do not undergo surgery before the disease has already progressed.

In contrast, testing for liquid-based biomarkers is not invasive and can facilitate preoperative diagnosis. While clinically validated tests have been approved as companion diagnostics for poly(ADP)ribose polymerase (PARP) inhibitors in OC and other tumor types in the US (9), currently, CA-125 is the only blood-borne marker recommended for the diagnosis and management of OC, which has been validated in numerous studies (10). Despite this, serum CA-125 levels cannot accurately discriminate benign from malignant ovarian lesions in premenopausal women (11), and CA-125 testing has low sensitivity in early disease stages (12), and does not provide detailed molecular information about the tumor. In addition, previous randomized clinical trials have not indicated a significant reduction in mortality from OC when screening using CA-125 level testing (13, 14). Hence, the US Preventive Services Task Force discourages the use of serum CA-125 levels to screen for OC (15). However, as early detection of OC is potentially cost-effective and may still improve survival (14, 16, 17), novel non-invasive strategies for early detection are in development and are urgently needed.

Broadly, liquid biopsies (LB) involve the analysis of cancer markers released by tumors in easily accessible bodily fluids, such as blood. These markers may include circulating tumor cells (CTCs), circulating tumor DNA (ctDNA), cell-free DNA (cfDNA) and exosome content such as microRNA (miRNA). CTCs have a low concentration in peripheral blood and specialized methods for their isolation and analysis in OC have been developed (18). In contrast, ctDNA/cfDNA has a relatively high concentration in peripheral blood and is detected using techniques such as digital droplet polymerase chain reaction (PCR), quantitative PCR, and next-generation sequencing (NGS) (18). By detection of molecular markers released by or present in cancer cells, LB retains the valuable insights into the molecular profile of the disease (e.g. homologous recombination deficiency [HRD]) afforded by tissue biopsy (19, 20), unlike CA-125 levels. The non-invasive nature of LB means that they are associated with less risk, patient pain, and potentially less cost, while being more easily repeatable than standard tissue biopsy (19, 20). These characteristics of LB may be of particular interest in China, where large regional variances and clusters of incidence and mortality are observed (21).

**Figure 1:**
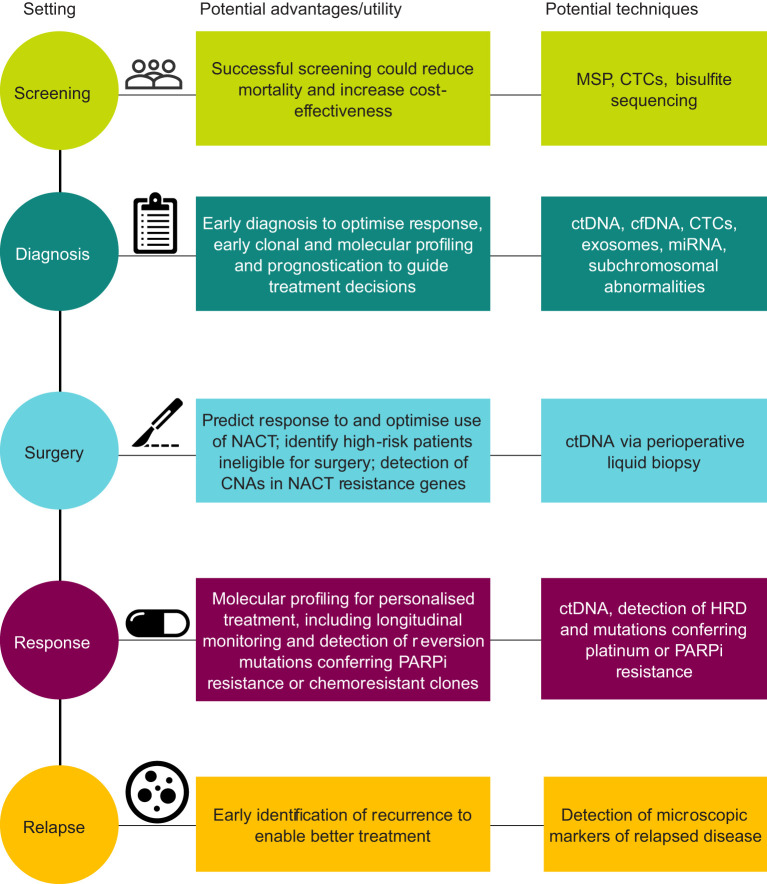
Schematic summary of liquid biopsy in ovarian cancer. CNA, copy-number alteration; CTC, circulating tumor cell; cfDNA, cell-free DNA; ctDNA, circulating tumor DNA; HRD, homologous recombination deficiency; miRNA, microRNA; MSP, methylation-specific PCR; NACT, neoadjuvant chemotherapy; PARPi, poly(ADP-ribose) polymerase inhibitor.

Here, we comprehensively review the use of LB for the diagnosis of OC, as well as for predicting patient outcomes, response to treatment, and disease progression (**Figure 1**).

We aim to increase awareness of the clinical relevance of LB in OC and thereby increase clinical adoption to improve early diagnosis and treatment outcomes, and call for future research on the identification of OC biomarkers in LB.

## 2 Screening

Developing better screening strategies may increase the rates of tumor detection at pre-symptomatic stages and improve outcomes. Ideal screening assays should be specific, sensitive, non-invasive, and cost-effective to enable adoption into routine clinical practice.

Most OC screening strategies using LB in pre-symptomatic individuals are based on cancer-specific epigenetic signatures detected in ctDNA or cfDNA isolated from blood (**Table 1**).

**Table 1:** Summary of studies using liquid biopsy to diagnose OC.

Tumor stage	n	Biopsy source	Laboratory method	Genetic marker	AUC (95% CI)	Detection rate, %	Specificity, % (95% CI)	Sensitivity, % (95% CI)	Ref.
cfDNA									
Methylation									
Stage III–IV OC	30	Plasma	Microarray	*RASSF1A, CALCA*, and *EP300*	NR	NR	86.7 (66.7–96.7)	90.0 (76.7–100)	(22)
Stage I–IV OC	87 (stage I, n = 41; stage II–IV, n = 46)	Serum	Methylation-specific PCR	*APC*, *CDH1*, *OPCML*, *RASSF1A*, *RUNX3*, *SFRP5*, and *TFPI2*	Overall: 0.9126 (0.8643–0.9609) Early stage: 0.8916 (0.8258–0.9574)	NR	90.57	89.66	(23)
Stage I–IV OC	47	Plasma	Methylation-specific PCR	*RASSF2A*	NR	51.1	NR	NR	(24)
Stage I–IV OC	43	Serum	Reduced-representation bisulfite sequencing	*COL23A1*, *C2CD4D*, and *WNT6*	NR	57.9 (34.0–78.9)^a^	88.1 (77.3–94.3)^a^	60.0 (27.4–86.3)^a^	(25)
Stage I–IV OC	194	Serum	Methylation-specific PCR	*OPCML*, *TFPI2*, and *RUNX3*	NR	NR	90.14	91.87	(26)
Stage I–IV (multiple tumors)	4077 (OC n=65)	Plasma	cfDNA bisulfite conversion and sequencing with ML	Methylation signatures from WGBS	NR	NR	99.5 (99.0–99.8)	Overall: 51.5 (49.6–53.3) OC: 83.1	(27)
Chromosomal/structural alterations									
Stage I–IV OC	32 (16 stage I–II, 16 stage III–IV)	Plasma	Low-coverage WGS	Subchromosomal abnormalities	NR	Overall: 40.6 (23.7–59.4) Early stage: 38	93.8 (79.2–99.2)	40.6 (23.7–59.4)	(28)
Stage I–IV OC	68 (57 with ovarian carcinomas, 11 with benign tumors)	Plasma	Low-coverage WGS	Chromosomal instability	Overall: 0.89 HGSOC: 0.94	NR	91	74	(29)
Other									
Stage III–IV OC	46	Serum	Tagged-amplicon deep sequencing	NR	NR	NR	97.5	97.5	(30)
ctDNA									
Mutations									
Stage II–III OC	21	Serum	NGS	*TP53* and *BRCA1*	NR	NR	100.0	73.7	(31)
Stage I–IV OC	96	Plasma	NGS	27 cancer-related genes	NR	Stage I: 50 Stage III: 46.2 Stage IV: 83.3	NR	Stage I: 43 Stage II: 73	(32)
Methylation									
Stage I–IV OC	26	Serum	Methylation-specific PCR	*SFRP1*, *SOX1*, and *LMX1A*	NR	NR	75	73	(33)
Stage I–IV OC	33	Plasma	Microarray	*HIC1, PAX5, BRCA1, PGR*, and *THBS1*	NR	NR	61.1	85.1	(34)
Stage I–IV OC	106	Serum	Methylation-specific PCR	*RASSF1A*	NR	51	NR	NR	(35)
Stage I–IV OC	36	Serum	Methylation-specific PCR	*SLIT2*	NR	80.6	NR	NR	(36)
Stage I–IV OC	49	Plasma	Pyrosequencing-based	*CDH1* and *PAX1*	0.932	NR	56	91	(37)
Stage I–IV OC	70	Serum	RT-PCR	*HOXA9* and *HIC1*	0.95	NR	100	88.9	(38)
CTCs									
Stage I–IV OC	129	Plasma	CAM-based cell enrichment, IHC	EpCAM, CA-125, CD44, separase	NR	Overall: 88.6 Stage I/II: 41.2	95.1	83	(39)
Stage I–IV OC	123	Plasma	Flow cytometry	NR	NR	85.3	97	83	(40)
Stage I–IV OC	109	Serum	Immunomagnetic bead screening with multiplex RT-PCR	EpCAM, *HER2, MUC1, WT1, P16, PAX8*	NR	Overall: 90 Stage I/II: 93	NR	NR	(41)
Stage I–IV OC	30	Serum	Microfluidic isolation and immunofluorescent staining	CD45, HE4, and epithelial and mesenchymal markers	0.716	73.3	63.0	73.3	(42)
Stage I–IV OC	160	Serum	Immunomagnetic bead screening with multiplex RT-PCR	EpCAM, *MUC1*, and *WT1*	0.893	Stage I/II: 74.5	92.2	79.4	(43)
Stage I–IV OC	22	Serum	Microfiltration with morphological and immunofluorescence analyses	EMT markers	NR	40.9	NR	NR	(44)
Exosomes/exosomal miRNAs									
Stage I–IV OC	78	Plasma	Nanoparticle tracking, ELISA	NR	NR	100	NR	NR	(45)
Stage III–IV OC	40	Plasma	LC-MS/MS, nanoparticle tracking, dynamic light scattering, TEM	LPB, FGG, FGA, GSN	GSN: 0.8309 (0.7343–0.9274) FGA: 0.8459 (0.7602–0.9317) FGG: 0.7447 (0.6323–0.8571) LBP: 0.6588 (0.5381–0.7794)	NR	NR	NR	(46)
EOC	55	Plasma	smRNA sequencing; RT-PCR	miR-4732-5p	AUC: 0.889	NR	85.7	82.4	(47)
Circulating miRNAs									
Stage III–IV OC	168	Serum	Microarray analysis, RT-PCR	miR-1246	0.89	NR	77	87	(48)

AUC, area under the receiver operating characteristic curve; CAM, cell adhesion matrix; cfDNA, cell-free DNA; CI, confidence interval; ctDNA, circulating tumor DNA; CTCs, circulating tumor cells; ELISA, enzyme-linked immunosorbent assay; EMT, epithelial-to-mesenchymal transition;IHC, immunohistochemistry; LC-MS/MS, liquid chromatography with tandem mass spectrometry; NGS, next-generation sequencing; NR, not reported; OC, ovarian cancer; PCR, polymerase chain reaction; RT, reverse transcriptase; smRNA, small messenger RNA; TEM, transmission electron microscopy; WGS, whole-genome sequencing.

a Within two years of sample collection.

Several studies conducted in China have attempted to evaluate the utility of LB for the detection of OC. For example, Dong et al. (36) found that the tumor suppressor gene *SLIT2* was hypermethylated in 29 of 36 (80.6%) Chinese patients with OC, but not in any of the 25 healthy women evaluated. In 27 of the 29 (93.1%) patients with tumor *SLIT2* hypermethylation, *SLIT2* was also aberrantly methylated in ctDNA samples. In a similar study in China, Wang et al. (26) used methylation-specific polymerase chain reaction (MSP) to analyze aberrantly methylated genes in cfDNA from 194 patients with OC, and found that *OPCML* was hypermethylated in patients with early-stage OC but not in healthy donors. Interestingly, serum levels of CA-125 did not differ between patients with OC and healthy donors (26). Aberrant methylation of *RASSF2A* in cfDNA was also observed in approximately 36% of plasma samples from patients with OC, but was not observed in patients with benign ovarian tumors or healthy volunteers (24).

Zhang et al. (23) developed a multiplex MSP assay for the detection of early-stage OC using serum cfDNA in China. The assay was based on seven genes that are frequently hypermethylated in OC: *APC*, *CDH1*, *OPCML*, *RASSF1A*, *RUNX3*, *SFRP5*, and *TFPI2*. Using preoperative cfDNA samples from 87 patients with OC (stage I, n = 41; stage II–IV, n = 46), 53 with benign ovarian tumors, and 62 healthy donors, the high specificity (90.5%) and sensitivity (85.3%) of this assay was notably higher than the respective values for CA-125 in this cohort (64.2% and 56.1%, respectively) (23).

More recently, results from the US/Canada-based Circulating Cell-free Genome Atlas study (CCGA) have been reported, which used a methylation-based cfDNA approach combined with machine learning to screen for multiple tumor types (27). With a high specificity of 99.5%, the test had an overall sensitivity of 51.5% across tumor types. Among patients with OC, the test had a sensitivity of 80.0–94.7% in patients with stage II–IV disease and 50.0% in patients with stage I disease (27).

While these early results from methylation-based screening are promising, further study is needed to further characterize and refine screening methods and drive more widespread and standard selection of genes of interest. Because of the relatively low prevalence of OC, screening assays need to demonstrate a high predictive value; hence, larger studies are needed to confirm that LB-based assays exhibit high specificity (>99.7%) and sensitivity (>75%) before adoption into routine clinical practice (49).

## 3 Early diagnosis

### 3.1 ctDNA and cfDNA

Tumor-specific genetic alterations can be detected by cfDNA and ctDNA, which are small DNA fragments released by apoptotic or tumor cells that circulate through the bloodstream (**Table 1**).

In one of the first studies involving sequencing of entire genes to detect cancer mutations in cfDNA, Forshew et al. (30) used tagged-amplicon deep sequencing (Tam-Seq) to screen nearly 6000 genomic regions for mutations in the plasma of patients with advanced (stage III–IV) OC. This non-invasive method allowed the identification of cancer mutations with frequencies as low as 2%, providing a sensitivity and specificity of 97.5%. This method also allowed the monitoring of the evolution of tumors over time and identification of the source of metastatic relapse in patients with multiple primary tumors (30).

A NGS analysis of tumor and plasma samples from 96 patients with OC showed that tumor somatic variants in at least one of 27 cancer-related genes were present in the serum of 83.3% of patients with stage IV OC; however, the sensitivity of this test was lower for early-stage disease (32). Mutations in *TP53* and *BRCA1* in ctDNA or cfDNA have also been shown to have diagnostic utility in OC (31), and analysis of Chinese patients has shown that mutation frequency in ctDNA using hybrid capture-based genomic profiling were generally similar between tissue biopsies and LB (50).

Multiple studies have shown that testing cfDNA or ctDNA samples for methylation of various genes, including *RASSF1A, CALCA, EP300, APC, CDH1, OPCML, RUNX3, SFRP5, COL23A1, C2CD4D, WNT6, TFPI2, HOXA9*, and *PAX1*, may help detect early-stage OC (22, 23, 25, 26, 37, 38).

Testing for chromosomal instability in cfDNA or ctDNA may help identify patients with early-stage ovarian tumors. In a proof-of-concept study, Vanderstichele et al. (29) conducted low-coverage whole-genome sequencing of plasma cfDNA from 68 patients with an adnexal mass, 57 of whom were diagnosed with OC. Chromosomal instability levels in cfDNA matched those in tissue biopsies and were significantly higher in patients with OC than in those with benign tumors or healthy individuals. Chromosomal instability testing in cfDNA detected OC with area under the curve (AUC) values of 0.89 in the entire cohort and 0.94 in patients with high-grade serous OC. These AUC values were higher than those of serum CA-125 (AUC=0.78) (29).

A prospective study involving low-coverage sequencing of preoperative samples of circulating DNA from 32 women with OC (16 stage I–II, 16 stage III–IV) and 32 women with benign tumors supports the potential utility of genomic aberrations in cfDNA to detect malignant tumors (28). Subchromosomal abnormalities in cfDNA were present in 13 of 32 (41%) patients with OC, compared with 2 of 32 women with benign neoplasms, leading to a specificity of 93.8% but sensitivity of 40.6%, suggesting that further refinement of these methods is required to improve their performance.

Liang et al. investigated differentially methylated regions in OC ctDNA from the Chinese Academy of Medical Sciences Hospital, and developed two models: one for detection and one for prognostication of OC (51). The detection model was superior to CA-125-based detection (AUC, 0.987 [95% CI, 0.971−1.00] *vs*. 0.940 [95% CI: 0.895−0.985]), and the prognostic model for risk stratification also outperformed CA-125 (AUC, 0.949 [95% CI: 0.85−1.00] *vs* AUC, 0.659 [95% CI: 0.44−0.87]). These encouraging improvements over CA-125-based detection and prognostication warrant further investigation.

### 3.2 CTCs

CTCs are tumor cells that have entered the peripheral blood from the original tumor. As such, CTCs may provide information on multiple facets of OC, such as molecular classification to enable risk stratification (52–54). However, the concentration of CTCs in peripheral blood in early stages of OC is low, necessitating specialized techniques for enrichment and detection (42, 54–56). These techniques may include immunoaffinity and immunomagnetic techniques (54), dialectrophoresis and other microfluidic techniques (44, 57), as well as others for enrichment and detection.

Despite requirement of these specialized techniques, CTCs have shown promise as diagnostic biomarkers for OC (**Table 1**), as highlighted by multiple Chinese studies. Zhang et al. (41) used immunomagnetic detection of epithelial antigens (EpCAM, HER2, and MUC1) for enrichment combined with multiplex reverse transcriptase-polymerase chain reaction (RT-PCR) to detect CTCs in serum samples from 109 patients with OC; CTCs were found in 98 (90%) patients.

In a prospective analysis of samples from 61 women with suspected OC in China, Guo et al. (42) used size-based microfluidic separation and immunocytochemical detection and found that the counts of CTCs expressing HE4 and epithelial-to-mesenchymal transition (EMT) markers without CD45 were significantly higher in patients diagnosed with OC than in those with benign lesions, providing 86.7% specificity in patients with CA-125 ≥35 U/mL (42). The sensitivity of CTCs for detecting OC was higher than that of plasma CA-125 levels (73.3% *vs*. 56.7%).

To further improve the diagnostic utility of CTCs in ovarian cancer, Wang et al. (43) developed an optimized detection method based on EpCAM, MUC1, and WT1. This method was highly specific (92.2%) and had 79.4% sensitivity. Notably, the detection rate of CTCs was higher than that of CA-125 for early-stage (stage I/II) tumors (74.5% *vs*. 58.2%, *P* = 0.069).

While these findings are promising, a key challenge limiting the clinical utility of CTC-based diagnostics in early stages of OC is the low number of CTCs in the early stages of the disease (42, 55, 56), as well as reported detection rates varying from 12 to 90% across different platforms (42). In contrast, CTCs can be found in higher numbers in the circulation of patients with advanced disease (stages III and IV), with diagnostic sensitivity and specificity reaching 76%–83% and 55%–97%, respectively (39–41, 58, 59).

### 3.3 Exosomes and miRNAs

Exosomes are small (30–100 nm) vesicles released by cells that regulate cellular communication and transfer of molecules, including RNA, DNA, and proteins. Exosomes released by cancer cells can be used as diagnostic markers (**Table 1**).

Zhang et al. found that exosomes from Chinese patients with OC were enriched in proteins involved in tumorigenesis and metastasis (46). Exosomal FGA and GSN levels were significantly elevated, whereas FGG and LBP levels were significantly downregulated in exosomes from Chinese patients with OC compared with those from healthy donors (46), providing proof-of-concept evidence that proteomic profiling of exosomes can be used to diagnose OC in Chinese patients.

Emerging evidence suggests that circulating miRNAs may serve as diagnostic markers for OC (**Table 1**). Todeschini et al. (48) analyzed serum samples from 168 patients with stage III–IV OC and 65 healthy volunteers. They found that the levels of miR-1246, miR-595, and miR-2278 were significantly higher in serum samples from patients with OC than those from healthy controls. Receiver operating characteristic curve analysis revealed that among these miRNAs, miR-1246 had the highest diagnostic utility, and had an AUC of 0.89, sensitivity of 87%, specificity of 77%, and diagnostic accuracy of 84%. In a similar study, Liu et al. (47) seven exosome-derived miRNAs (miR-4732-5p, miR-877-5p, miR-574-3p, let-7a-5p, let-7b-5p, let-7c-5p, and let-7f-5p) were up-regulated and two down-regulated (miR-1273f and miR-342-3p) in patients with EOC; miR-4732-5p had an AUC of 0.889, with 85.7% sensitivity and 82.4% specificity in diagnosis of EOC. Another exploratory study in Chinese patients found that exosomal miRNA-205 expression was significantly associated with OC, and had elevated levels during metastasis (60), and exploratory analysis of circular RNAs in Chinese patients found that such RNAs may have diagnostic utility in combination with CA-125 (61). While a range of miRNAs have been identified as potential OC biomarkers (62), the heterogeneity of OC means that more studies are needed to assess the diagnostic utility of circulating and exosomal non-coding RNAs in patients with OC so that clearer and more consistent miRNA signatures and profiles can be developed and allow more routine early diagnosis using LB.

## 4 Surgery/perioperative liquid biopsy

Following diagnosis of OC, an early treatment decision is whether initial cytoreductive surgery should be primary (upfront) or interval (i.e. following neoadjuvant chemotherapy [NACT]). While large randomized trials have generally not found significant differences in survival outcomes between the two approaches (63–66), SGO/ASCO guidelines recommend that this decision is made according to clinical risk to avoid unnecessary exposure to platinum-based chemotherapy (67). In this way, LB represent a valuable tool in risk stratification by providing a non-invasive method that enables early identification of factors before surgery that may predict response to NACT such as platinum resistance, prognostic factors following surgery such as microscopic residual disease, and monitor response to treatment to guide treatment decisions.

Mutations in post-surgical ctDNA have been associated with inferior survival outcomes (68), detection of post-surgical ctDNA outperforms CA-125 monitoring as a predictor for mortality (69, 70), may be predictive of complete resection following NACT or following surgery (70, 71), and copy number alterations in *MROH1*, *TMEM249*, and *HSF1* in ctDNA of patients with OC resistant to NACT were significantly associated with worse OS and high expression levels compared with patients with NACT-sensitive disease, suggesting that specific ctDNA mutations could be useful in LB for response monitoring and prediction (72). Larger, prospective studies of risk stratification and biomarker identification using perioperative LB are warranted to enable routine clinical adoption.

## 5 Treatment response and monitoring progression

### 5.1 Predicting and monitoring response to PARP inhibition

PARP (poly-ADP-ribose polymerase) inhibitors prevent repair of single-stranded breaks in DNA, generating double-stranded breaks that cannot be accurately repaired in tumors with HRD (73). HRD is typically caused by germline or somatic *BRCA1*/*BRCA2* mutations, epigenetic factors such as *BRCA1*/*BRCA2* silencing via promoter methylation, or potentially other genetic or genomic causes of genomic instability such as telomeric allelic imbalance, loss of heterozygosity, or large-scale state transitions in OC and other tumor types (74–77).

The efficacy of PARP inhibition (with or without bevacizumab) for OC has been demonstrated in global clinical trials (78–82), particularly as first-line maintenance therapy. Several PARP inhibitors have been approved in China for the treatment of newly diagnosed advanced HRD-positive, or platinum-sensitive relapsed OC and emerging real-world evidence highlights the importance of HRD as a biomarker to predict response to PARP inhibition in China (83–85). Based on results from the global phase III PRIMA trial, the PARP inhibitor niraparib was approved in China for patients with newly diagnosed advanced HRD-positive or HRD-negative tumors (86), though the benefit in PFS was most pronounced among patients who had HRD-positive tumors (median PFS for niraparib *vs* placebo among patients with HRD-positive tumors, 24.5 *vs* 11.2 months; hazard ratio [HR], 0.52 [95% CI, 0.40–0.68] and for patients with HRD-negative tumors 8.4 *vs* 5.4 months; HR, 0.65 [95% CI, 0.49–0.87]) and the higher, 300 mg, starting dose (78, 87, 88). Multiple LB have been approved as companion diagnostics for PARP inhibitors in various indications, including to detect HRD in OC and prostate cancer (9). Therefore, various studies have been conducted to assess the value of LB as a non-invasive method to assess HRD status and predict response to PARP inhibition (**Table 2**).

**Table 2:** Summary of studies using liquid biopsy to predict or monitor response to treatment in patients with ovarian cancer (n≥10).

Tumor subtype and stage	n	Specimen	Laboratory method	Genetic marker	Treatment	Outcome or Clinical application	Ref.
ctDNA or cfDNA							
Mutations							
Stage I–IV EOC	137	Plasma	DNA sequencing, PCR	*TP53*	PBC	Response monitoring	(89)
Relapsed HGSOC	40	Plasma	Microfluidic digital PCR	*TP53*	Chemotherapy (PBC or not)	Response monitoring	(90)
PSR HGSOC	18	Plasma	NGS	*TP53*	PARP inhibitor (rucaparib)	Response monitoring	(91)
Stage II–IV HGSOC	102	Plasma	ddPCR	*TP53*	Platinum–taxane	Response monitoring	(92)
Stage I–IV HGSOC	30	Plasma	NGS	*BRCA1*/*BRCA2* reversion	PBC and PARP inhibitor	Treatment resistance	(93)
Stage III–IV HGSOC	19	Plasma	NGS	*BRCA1*/*BRCA2* reversion	PARP inhibitor	Resistance	(94)
Stage I–IV ovarian cancer	121	Plasma	NGS	Pathogenic germline or somatic *BRCA1*/*BRCA2*	PARP inhibitor	Sensitivity/response	(95)
HGSOC	97	Plasma	NGS	*BRCA1*/*BRCA2* reversion	PARP inhibitor (rucaparib)	Primary and acquired resistance	(96)
Stage III–IV HGSOC	38	Serum	Tagged-amplicon deep sequencing	Mutations in *TP53, PTEN, BRAF, KRAS, EGFR*, and *PIK3CA*	PBC	Response monitoring	(30)
Stage I–IV ovarian clear cell carcinoma	29	Plasma	ddPCR	Mutations in *KRAS* and *PIK3CA*	PBC	Response monitoring	(97)
Stage III–IV HGSOC	14	Plasma	NGS/Ion Torrent panel	Ion Torrent panel genes	Neoadjuvant PBC	Response monitoring	(98)
Methylation							
Stage I–IV EOC	43	Serum	Reduced representation bisulfite sequencing	*COL23A1*, *C2CD4D*, and *WNT6*	PBC	Response monitoring	(25)
Stage I–IV HGSOC	50	Plasma	High-resolution melting analysis	*ESR1* promoter	PBC	Treatment resistance	(99)
Platinum-resistant *BRCA*-mutated ovarian cancer	32	Plasma	Methylation-specific ddPCR	*HOXA9* promoter	PARP inhibitor (veliparib)	Resistance	(100)
Stage I–IV recurrent ovarian cancer	126	Plasma	Methylation-specific ddPCR	*HOXA9* promoter	Chemotherapy followed by maintenance therapy with PARP inhibitors or bevacizumab	Resistance	(101)
Other							
Stage II–IV HGSOC	12	Plasma	NGS	*ERBB2* amplification	PBC ± trastuzumab	Response monitoring	(102)
Stage I–IV ovarian cancer	11	Serum	RT-PCR	ctDNA level	Chemotherapy or PARP inhibitor	Increase in ctDNA levels after the first treatment cycle is associated with response	(103)
CTCs							
Stage I–IV ovarian cancer	143	Plasma	Immunomagnetic CTC enrichment, multiplex RT-PCR	ERCC1+ CTCs	PBC	Treatment resistance	(104)
Stage I–IV ovarian cancer	65	Plasma	AdnaTest Ovarian Cancer, multiplex RT-PCR	ERCC1+ CTCs	PBC	Treatment resistance	(105)
Stage I–IV ovarian cancer	54	Serum	Nanoroughened microfluidic-based enrichment	EpCAM+, DAPI+, CD45–	PBC	Treatment resistance	(106)
Stage I–IV EOC	160	Serum	Immunomagnetic bead screening with multiplex RT-PCR	MUC1+ CTCs	PBC	Treatment resistance	(43)
Exosomes							
Stage I–IV EOC	78	Plasma	Nanoparticle tracking analysis, ELISA	Exosomal HLA-G	PBC	Treatment resistance	(45)

cfDNA, cell-free DNA; ctDNA, circulating tumor DNA; CTCs, circulating tumor cells; ddPCR, droplet digital PCR; ELISA, enzyme-linked immunosorbent assay; EMT, epithelial-to-mesenchymal transition; EOC, epithelial ovarian carcinoma; HGSOC, high-grade serous ovarian cancer; IHC, immunohistochemistry; NGS, next-generation sequencing; NR, not reported; PBC, platinum-based chemotherapy; PCR, polymerase chain reaction; RT-PCR, reverse-transcriptase PCR.

#### 5.1.1 cfDNA and ctDNA

Ratajska et al. (95) used NGS to analyze ctDNA samples from 121 patients with stage I–IV OC, demonstrating that 30 of the 121 (24.8%) patients had ctDNA with pathogenic germline or somatic *BRCA1*/*BRCA2* mutations, comparable to reported germline and somatic BRCA mutation prevalences in China (107–109) and providing proof-of-concept evidence that *BRCA1*/*BRCA2* mutation testing using ctDNA samples from patients with OC could be used in the clinic to identify patients best suited for PARP inhibition and to monitor response.

Reversion mutations in *BRCA1*/*BRCA2* that restore protein function have been associated with the development of resistance to PARP inhibitors. In an NGS analysis of pretreatment ctDNA samples from 96 patients with OC, Lin et al. (96) found that *BRCA1*/*BRCA2* reversion mutations in ctDNA were associated with primary and acquired resistance to rucaparib. NGS studies of preoperative cfDNA samples from patients with OC harboring germline *BRCA1*/*BRCA2* mutations showed that reversion alterations restoring the *BRCA1/BRCA2* open reading frame (ORF) were associated with resistance to PARP inhibition in patients with recurrent disease (93, 94), and that these reversion mutations may be caused by the microhomology-mediated end joining pathway (110).

Rusan et al. (100) found that the methylation levels of *HOXA9* in ctDNA during treatment were associated with poor response to PARP inhibition in patients with platinum-resistant, *BRCA1*/*BRCA2*-mutated OC. Survival outcomes were significantly inferior in patients with detectable *HOXA9* methylation in ctDNA than in those without *HOXA9* methylation (median progression-free survival [PFS]: 5.1 *vs*. 8.3 months, *P* < 0.0001; median OS: 9.5 *vs*. 19.4 months, *P* < 0.002) (100). Faaborg et al. reported similar findings in a study of 126 patients (38.9% platinum sensitive and 81.7% with recurrent OC undergoing treatment with chemotherapy followed by maintenance therapy with PARP inhibitors or bevacizumab (101); with the increasing use of PARP inhibition in earlier lines of therapy, validation of these biomarkers in first line will be increasingly important.

*TP53* mutations are one of the most prevalent genetic alterations in OC. In the Phase II ARIEL2 study of rucaparib in platinum-sensitive relapsed OC, targeted amplicon deep sequencing to detect low-frequency mutations in *TP53* in ctDNA suggested that reduction in the frequency of *TP53* mutations in ctDNA during treatment was associated with response to rucaparib (91).

### 5.2 Identifying platinum resistance and monitoring progression

#### 5.2.1 cfDNA and ctDNA

Evaluating genetic markers of response to chemotherapy using LB is emerging as a promising approach for molecular profiling in patients with OC. As LB are easy to obtain and repeatable, they may be used for longitudinal monitoring of treatment response and disease progression (**Table 2**).

To evaluate the clinical utility of cfDNA analysis to monitor response to chemotherapy and disease progression, Arend et al. (98) conducted NGS analysis of cfDNA samples, which provided proof-of-concept evidence that cfDNA analysis before and after treatment can be used to monitor disease progression and the genetic evolution of tumors during chemotherapy.

In an analysis of pretreatment ctDNA samples from patients with OC, Lin et al. (96) found that *BRCA1*/*BRCA2* reversion mutations were significantly more frequent in patients with platinum-refractory (18%; 2/11) or platinum-resistant (13%; 5/38) disease compared with platinum-sensitive disease (2%; 1/48). Another analysis of preoperative cfDNA samples from 30 patients with OC harboring germline *BRCA1* or *BRCA2* mutations showed that reversion alterations restoring the *BRCA1/BRCA2* ORF were associated with resistance to platinum-based chemotherapy in patients with recurrent disease (93).

A longitudinal analysis of ctDNA samples to assess for mutations in more than 500 cancer-related genes revealed good concordance of genetic alterations in ctDNA and tumor samples from 12 patients with OC (102). The study also showed that testing for *ERBB2* amplification in ctDNA from relapsed OC patients could identify patients who may benefit from ERBB2/HER2 inhibitors, such as trastuzumab (102).

In addition to mutations and structural aberrations in cfDNA or ctDNA, methylation of various genes, including *COL23A1*, *C2CD4D*, *WNT6, ESR1*, and *HOXA9*, has been shown to be associated with resistance to chemotherapy and could be used to predict or monitor treatment response (25, 99–101), suggesting that LB-based molecular testing may be useful in this setting, particularly in patients ineligible for tissue biopsy or for whom archival tissue is not available.

#### 5.2.2 CTCs

Data from various studies suggest that CTCs could be used as markers of response or platinum resistance in patients with OC. For example, the numbers of EpCAM-positive CTCs and MUC1-positive CTCs were significantly higher in chemoresistant patients than in patients who responded to chemotherapy (26.3% *vs*. 11.9%, *P* < 0.05; 26.4% *vs*. 13.4%, *P* < 0.05; **Table 2**) (43).

The number of ERCC1-positive CTCs has also been associated with chemotherapy resistance. CTC enrichment analyses in patients with OC showed that the presence of ERCC1-positive CTCs at diagnosis was a significant predictor of resistance to platinum-based chemotherapy (104, 105). Despite the predictive role of ERCC1-positive CTCs, ERCC1 expression levels in primary tumor tissues and circulating *ERCC1* mRNA levels did not predict resistance to chemotherapy, suggesting a particularly important role for LB in this setting.

Additionally, enrichment of CTCs (EpCAM-positive, DAPI-positive, and CD45-negative) using a nanoroughened microfluidic device showed that in 54 patients with stage I–IV OC, the number of CTCs was significantly associated with platinum resistance (106).

#### 5.2.3 Exosomes

Exosomes have been implicated in metastasis and treatment resistance in patients with OC. Au Yeung et al. (111) conducted preclinical NGS analysis of exosomes from cancer-associated adipocytes (CAAs), cancer-associated fibroblasts (CAFs), and OC cells. Interestingly, they found that the levels of miR21 were significantly higher in exosomes from CAAs and CAFs than in those from OC cells. They also found that miR-21 transfer from CAAs and CAFs to ovarian cancer cells resulted in *APAF1* silencing, thereby promoting chemoresistance and suppressing apoptosis (111). These findings suggest that levels of miR21 in exosomes may predict the risk of metastasis and chemoresistance in patients with OC, although clinical validation is required (**Table 2**).

Similar mechanistic studies have shown that exosomal miR-1246, miR-223, miR-183-5p, miR-130a, and miR-374a promote chemoresistance in OC (112–115). Additionally, Schwich et al. (45) showed that exosomal HLA-G levels were associated with platinum resistance. The clinical utility of these exosomal markers in OC requires further evaluation in clinical studies.

## 6 Prognostication and monitoring progression

### 6.1 cfDNA and ctDNA

Detection of cfDNA or ctDNA levels as well as examination of genetic and epigenetic characteristics are areas of great interest and have been well studied in the context of prognostication and monitoring of disease progression in OC (**Table 3**).

**Table 3:** Summary of studies using liquid biopsy to predict outcomes in patients with ovarian cancer.

Tumor subtype and stage	n	Specimen	Laboratory method	Genetic marker	Setting	Outcome prediction	Ref.
ctDNA or cfDNA							
Mutations							
Stage I–IV OC	10	Serum	ddPCR	Tumor-specific	Relapsed disease	Poor OS (*P* = 0.0194) and PFS (*P* = 0.0011)	(70)
Stage I–IV OC	11	Plasma	ddPCR	Tumor-specific	After debulking surgery	Early recurrence detection; tumor volume following recurrence	(116)
Stage I–IV EOC	137	Plasma	DNA sequencing, PCR	*TP53*	NR	Poor OS (*P* = 0.02)	(89)
Relapsed HGSOC	40	Plasma	Microfluidic digital PCR	*TP53*	Chemotherapy	TTP (HR: 0.22 [95% CI, 0.07–0.67], *P* = 0.008)	(90)
Stage II–IV HGSOC	102	Plasma	ddPCR	*TP53*	PBC	TTP (*P* = 0.038)	(92)
HGSOC	97	Plasma	NGS	*BRCA1*/*BRCA2* reversion	PARP inhibitor (rucaparib)	Poor PFS (HR: 8.33, *P* < 0.0001)	(96)
Methylation							
Stage I–IV HGSOC	59	Plasma	Methylation-sensitive high-resolution melting analysis	*RASSF1A* promoter	Platinum-based chemotherapy	Poor OS (HR: 2.76 [95% CI, 1.102–6.915], *P* = 0.030)	(117)
Platinum-resistant *BRCA*-mutated ovarian cancer	32	Plasma	Methylation-specific ddPCR	*HOXA9* promoter	Treatment with PARP inhibitor (veliparib)	Poor OS (*P* < 0.002) and PFS (*P* < 0.0001)	(100)
Stage I–IV recurrent ovarian cancer	100	Plasma	Methylation-specific ddPCR	*HOXA9* promoter	Chemotherapy followed by maintenance therapy with PARP inhibitors or bevacizumab	Poor OS (HR: 2.17 [1.18–3.98]; *P* = 0.013)	(101)
Other							
Stage I–IV OC	164	Plasma	RT-PCR	cfDNA ≥ 22,000 IU/mL	Before surgery	Poor DFS (multivariate HR, 2.22 [1.16–4.21]; *P* = 0.01)	(118)
Stage I–IV EOC	36	Serum	RT-PCR	*RAB25* downregulation	Before surgery	Poor OS (HR: 33.6 [95% CI, 1.8–634.8], *P* = 0.02) and DFS (HR: 18.2 [95% CI, 2.0–170.0], *P* = 0.01)	(119)
Stage I–IV EOC	63	Serum	PCR-based fluorescence microsatellite analysis	LOH at 6q and 10q	Before surgery and after chemotherapy	OS (*P* = 0.030)	(120)
CTCs							
Stage I–IV EOC	90	Peripheral blood	Immunomagnetic assay	MOC-31+ CTCs	Prior to adjuvant chemotherapy	No association with prognosis	(121)
Stage I–IV EOC	64	Peripheral blood	Immunocytochemistry	NR	Prior to debulking surgery	No association with prognosis	(122)
Stage I–IV EOC	71	Peripheral blood	Immunomagnetic CTC enrichment	Cell adhesion matrix molecules and epithelial markers	NR	Poor disease-free survival (*P* = 0.042)	(123)
Stage I–IV EOC	122	Peripheral blood	Immunomagnetic enrichment	EpCAM, MUC-1, HER-2	At primary diagnosis and/or after platinum-based chemotherapy	Poor OS before surgery (*P* = 0.0054) and after chemotherapy (*P* = 0.047)	(124)
Stage I–IV EOC	216	Peripheral blood	CTC enrichment	EpCAM+, cytokeratin+, CD45−	Platinum-based chemotherapy	Poor PFS (HR: 1.58 [95% CI, 0.99–2.53], *P* = 0.0576) and OS (HR: 1.54 [95% CI, 0.93–2.54], *P* = 0.0962)	(55)
Stage I–IV EOC	129	Plasma	CAM-based cell enrichment, IHC	EpCAM, CA-125, DPP4, CD44, seprase and cytokeratins	Before surgery	Poor OS (*P* = 0.0219) and PFS (*P* = 0.0024)	(39)
Stage I–IV EOC	143	Plasma	Immunomagnetic CTC enrichment, multiplex RT-PCR	ERCC1+ CTCs	Platinum-based chemotherapy	Poor OS (HR: 2.5 [95% CI, 1.1–5.5], *P* = 0.026) and PFS (HR: 3.4 [95% CI, 1.4–8.3], *P* = 0.009)	(104)
Stage I–IV EOC	123	Plasma	iCTC flow cytometry assay	Seprase and CD44	Before chemotherapy	Associated with relapse during and after treatment	(40)
Stage I-IV ovarian cancer	65	Plasma	AdnaTest Ovarian Cancer, multiplex RT-PCR	ERCC1	Platinum-based chemotherapy	Poor OS (*P* = 0.0008) and PFS (*P* = 0.0293)	(105)
Stage I–IV ovarian cancer	54	Serum	Nanoroughened microfluidic-based enrichment	EpCAM+, DAPI+, CD45–	Platinum-based chemotherapy	Poor PFS (HR: 1.3 [95% CI, 0.230–7.145], *P* = 0.035)	(106)
Stage I–IV ovarian cancer	266	Plasma	Density gradient centrifugation, immunostaining	EpCAM, EGFR, HER2, MUC1, cytokeratins, CD45	Samples collected at diagnosis and after first-line adjuvant first-line chemotherapy	Baseline CTC numbers associated with poor OS (HR: 3.305 [95% CI, 1.386–7.880], *P* = 0.007) and PFS (HR: 5.671 [95% CI, 1.560–20.618], *P* = 0.008)	(125)
Stage I–IV EOC	109	Serum	Immunomagnetic bead screening, RT-PCR	EpCAM+ CTCs, HER2+ CTCs	Platinum-based chemotherapy	Association with tumor stage (*P* = 0.034),	(41)
Stage III–IV HGSOV	46	Plasma	Shallow whole-genome sequencing	19p31.11 and 19q13.42 amplification	During platinum-based chemotherapy	Poor PFS (HR: 3.31 [95% CI, 1.33–9.13]; *P* = 0.011)	(126)
Stage I–IV ovarian cancer	1285	NR	Different enrichment methods	NR	Chemotherapy or surgery	Poor OS (HR: 1.77 [95% CI, 1.42–2.21], *P* < 0.00001) and PFS (HR: 1.53 [95% CI,1.26–1.86], *P* < 0.0001)	(127)
Stage I–IV EOC	160	Serum	Immunomagnetic bead screening combined with multiplex RT-PCR	EpCAM, MUC1, and WT1	Platinum-based chemotherapy	Poor OS (HR: 1.900 [95% CI, 1.020−3.540]; *P* = 0.043)	(43)
Exosomes							
Stage I–IV EOC	78	Plasma	Nanoparticle tracking analysis, ELISA	Exosomal HLA-G	Platinum-based chemotherapy	Poor PFS (HR: 1.8 [95% CI, 1.1–3.6]; *P* = 0.029)	(45)
Stage III–IV EOC	40	Plasma	Liquid chromatography-tandem mass spectrometry, nanoparticle tracking analysis, dynamic light scattering, transmission electron microscopy	LPB, FGG, FGA, GSN	NR	Poor OS and PFS	(46)
Circulating miRNAs							
Stage I–IV EOC	70	Serum	RT-PCR	miR-200a, miR-200b, miR-200c	NR	Expression levels of miR-200a and miR-200c were associated with disease progression (*P* = 0.04 and *P* < 0.001)	(128)
Stage I–IV EOC	207	Serum	TaqMan Low-Density Arrays, RT-PCR	miR-1274B, miR-200b, miR-141	Before treatment with bevacizumab plus chemotherapy	Low levels of miRNAs are associated with improved OS mir 1274B: HR = 0.846 (95% CI, 0.70–1.02); *P* = 0.085 miR 200b: HR = 0.798 (95% CI, 0.68–0.94); *P* = 0.006 miR-141: HR = 0.914 (95% CI, 0 0.81–1.03); *P* = 0.153	(129)

cfDNA, cell-free DNA; CI, confidence interval; ctDNA, circulating tumor DNA; CTCs, circulating tumor cells; ddPCR, droplet digital PCR; EMT, epithelial-to-mesenchymal transition; EOC, epithelial ovarian carcinoma; HGSOC, high-grade serous ovarian cancer; HR, hazard ratio; IHC, immunohistochemistry; NGS, next-generation sequencing; NR, not reported; LOH, loss of heterozygosity; OS, overall survival; PCR, polymerase chain reaction; PFS, progression-free survival; RT-PCR, reverse-transcriptase PCR; TTP, time to progression.

In analysis of pre-surgical cfDNA from patients with OC, Kamat et al. (118) found that higher levels of cfDNA (≥22,000 IU/mL) were significantly associated with worse survival, with multivariate analysis indicating that higher cfDNA levels were independently associated with worse disease-specific survival.

Hou et al. (69) found that when examining pre-surgical samples, ctDNA was more frequently detected and its levels significantly elevated in patients who subsequently experienced disease progression and died, with numerically higher ctDNA positivity and levels found in patients with high-grade OC. Following surgery, the presence of ctDNA was significantly associated with poor RFS, and all patients with ctDNA following surgery experienced disease progression; moreover, ctDNA-based methods detected recurrence 10 months before CT imaging (69). Similarly, Minato et al. (116) developed a droplet digital PCR-based assay to detect tumor-specific mutations in cfDNA in plasma, which was able to detect disease progression in all six patients who experienced disease recurrence. ctDNA levels were associated with increased tumor volume after recurrence. Notably, in both of these studies, analysis of ctDNA was able to detect disease recurrence earlier than CA-125 (69, 116).

Beyond cfDNA levels, genetic or epigenetic alterations in cfDNA or ctDNA associated with poor outcomes in patients with OC who receive PARP inhibitors or chemotherapy include *RAB25* downregulation [associated with poor OS in patients before surgery (119)], loss of heterozygosity at 6q and 10q [associated with poor OS in patients before surgery (120)], *HOXA9* promoter methylation [associated with poor OS and PFS in patients who received veliparib (100, 101)], *RASSF1A* promoter methylation [associated with poor OS following chemotherapy (117)], and *BRCA1*/*BRCA2* reversion mutations [associated with poor PFS in patients receiving rucaparib (96)]. Several studies have also shown that *TP53* mutations in plasma DNA were associated with shorter time to progression and poor OS (**Table 3**) (89, 90, 92).

### 6.2 CTCs and exosomes

The clinical significance of CTCs in OC is controversial (**Table 3**). Early studies showed no association between CTCs and survival outcomes (121, 122). However, Fan et al. defined invasive CTCs as those expressing CAM molecules and epithelial markers; the presence of these invasive CTCs in 71 patients with suspected OC was significantly associated with poor DFS (123), but had no significant impact on OS. In contrast, a similar study involving the detection of CTCs before surgery and after chemotherapy in 122 patients found that the presence of CTCs (based on EpCAM, MUC-1, and HER-2 expression) was associated with poor OS, but not DFS or PFS (124). Further research is needed to clarify the role of CTCs and the impact of specific molecular markers on outcomes in OC.

ERCC1 has also been proposed to predict poor outcomes among patients with OC; in 143 patients the presence of ERCC1-positive CTCs at diagnosis was a significant predictor of poor OS (HR, 2.5 [95% CI, 1.1–5.5]) and PFS (HR, 3.4 [95% CI, 1.4–8.3]) (104), with similar findings in another subsequent study (105).

In another study of patients with newly diagnosed OC, CTCs were detected in 98 of 109 (90%) patients (41). In this cohort, the number of CTCs was significantly associated with tumor stage (*P* = 0.034), and the expression of EpCAM and HER2 in CTCs was associated with chemoresistance (*P*=0.003 and *P*=0.035, respectively). The number of EpCAM-positive CTCs was significantly associated with poor OS (*P*=0.041).

Lee et al. (106) developed a nanoroughened microfluidic device that facilitates the enrichment of CTCs as EpCAM-positive, DAPI-positive, and CD45-negative circulating cells, and found that the number of CTCs was associated with worse PFS and platinum resistance.

Another study in patients with stage I–IV EOC showed that the presence of MUC1-positive CTCs was associated with poor OS; however, similar to previous reports, PFS was unaffected (43). A meta-analysis of data from two clinical trials and 13 retrospective studies involving 1285 patients found that the presence of CTCs was significantly associated with poor OS (HR: 1.77 [95% CI, 1.42–2.21], *P <*0.00001) and PFS (HR: 1.53 [95% CI, 1.26–1.86], *P <*0.0001) (127). A significant role of CTCs was observed across different clinical settings, including pre-treatment patients and patients undergoing debulking surgery. Notably, the predictive role of CTCs seemed to vary depending on the CTC enrichment method, which might explain the contradictory findings regarding the significance of CTCs in patients with OC.

Schwich et al. (45) found that exosomal HLA-G levels were significantly higher in patients with OC than in healthy donors. Although the total number of exosomes was not associated with outcomes, increased levels of exosomal HLA-G were associated with aggressive tumor features and poor outcomes, including residual tumor burden, high numbers of CTCs, and poor PFS.

### 6.3 Circulating miRNAs

Circulating miRNAs may be associated with outcomes in patients with OC (**Table 3**). Zuberi et al. (128) analyzed the expression levels of miR-200a, miR-200b, and miR-200c in the serum of patients with stage I–IV EOC, and found that the expression of miR-200a and miR-200c appeared to be associated with advanced disease stage and presence of metastasis. Similarly, Halvorsen et al. (129) assessed the levels of miR-1274B, miR-200b, and miR-141 in the serum of patients with OC and found that low levels of these miRNAs were associated with improved OS.

## 7 Conclusions

Accumulating evidence supports the diagnostic, predictive, and prognostic utility of multiple markers present in LB for OC, suggesting that LB potentially offers non-invasive, easily repeatable, accurate tools that may allow for early detection of OC and improve response prediction and early molecular profiling. Longer-term prospective studies, including cost-effectiveness analyses, are needed to assess the impact on patient outcomes. Such tools may be particularly useful among patients ineligible for surgery, who represent a notable proportion of patients with OC. However, widespread clinical implementation still faces many challenges. A key challenge is assay sensitivity and specificity for analysis of minute amounts of tumor-derived material. The accuracy of current diagnostic tests still needs to be improved. Another main challenge for current LB assays is the need for specialized equipment and technical expertise, which leads to long turnover time and high cost, making these assays inappropriate for routine clinical applications. The improvements to standardized and automated processing and analysis methods will help streamline workflow, ensure reliability and reproducibility, and reduce turnover time and cost. Moreover, many previous studies were limited by their sample sizes and designs, making their results difficult to interpret or reproduce. Longer-term prospective studies with appropriate designs, including cost-effectiveness analyses, are needed to assess the impact of LB on the outcomes of patients with OC. It is also noteworthy that most data exploring the clinical utility of LB for OC have focused on ctDNA and cfDNA; further research in larger, prospective studies regarding the clinical utility of other markers within LB for OC, such as exosomes, circulating non-coding RNAs, or to identify other markers is needed to further refine LB for clinical adoption.

## Author contributions

HZ: Conceptualization, Writing – review & editing. LW: Conceptualization, Writing – original draft. HW: Conceptualization, Writing – review & editing.
